# A rare case of multiple visceral vascular variations around the kidneys: morphological and clinical aspects

**DOI:** 10.1590/1677-5449.202301202

**Published:** 2024-02-12

**Authors:** Dibakar Borthakur, Mohammed Ahmed Ansari, Neerja Rani, Rajesh Kumar, Monica Baxla

**Affiliations:** 1 All India Institute of Medical Sciences – AIIMS, New Delhi, India.

**Keywords:** anatomical variation, kidney, renal vein, suprarenal, testicular artery, variação anatômica, rim, veia renal, suprarrenal, artéria testicular

## Abstract

Knowledge of the anatomical variations of the visceral branches of the abdominal aorta is important information for planning any surgeries in the region. We present here a rare constellation of variations of visceral vessels around the kidneys with a brief review of the recent literature. On the right side, an accessory renal artery was observed originating just distal to the main renal artery. The middle suprarenal artery was absent on the right side and there were two inferior suprarenal arteries originating from a branch of the main right renal artery. On the left side, the testicular artery had an arched course anterior to the left renal vein mimicking an unusual variety of nutcracker phenomenon. The right kidney was drained by two renal veins into the inferior vena cava. Knowledge of the coexistence of such complex anatomical variations might be helpful for clinicians during diagnostic and therapeutic procedures.

## Resumo

O conhecimento das variações anatômicas dos ramos viscerais da aorta abdominal é uma informação importante para o planejamento de qualquer cirurgia nessa região. Neste relato, apresentamos um raro conjunto de variações de vasos viscerais ao redor dos rins, bem como uma breve revisão da literatura recente. No lado direito, foi observada uma artéria renal acessória originando-se distal à artéria renal principal. Não havia artéria suprarrenal média no lado direito, e havia duas artérias suprarrenais inferiores originando-se de um ramo da artéria renal direita. No lado esquerdo, a artéria testicular apresentava um curso arqueado anterior à veia renal esquerda, simulando uma variedade incomum do fenômeno do quebra-nozes. O rim direito era drenado por duas veias renais para a veia cava inferior. O conhecimento da coexistência de tais variações anatômicas complexas pode ser útil para os clínicos durante os procedimentos diagnósticos e terapêuticos.

## INTRODUCTION

The terms ‘accessory renal artery’ and ‘aberrant renal artery’ are not synonymous. Any extra artery arising from the aorta and supplying the kidney is termed an ‘accessory renal artery’ whereas ‘aberrant renal artery’ indicates any extra artery supplying the kidney but arising from sources other than the aorta. Some authors have used ‘supernumerary renal artery’ to denote the same. Inconsistencies in the nomenclature prevail across the literature and the terms accessory renal artery, aberrant renal artery, and supernumerary renal artery are used interchangeably, though they are not the same.^1,2^ Satyapal et al.^3^ and Papaloucas et al.^4^ proposed that the term ‘additional’ for any artery in addition to the main renal artery be used to designate all different types of extra arteries supplying the kidneys. Some suggest that the term ‘aberrant renal artery’ should be reserved for those arteries supplying the kidney but not entering through the renal hilum. In this report, we use the term ‘accessory renal artery’ (ARA) for the unilateral variation observed. Occurrence of ARA is common, with a reported prevalence of around 25-30%.^5^ The renal arteries are paired lateral branches from the abdominal aorta arising approximately at the transpyloric plane just caudal to the origin of the superior mesenteric artery. ARA are frequently encountered distal to the main renal artery and can arise from the aorta and other arteries in the vicinity.^5,6^

The arterial supply to the suprarenal gland is peculiar and does not follow the typical hilar entrance route. The suprarenal gland is usually supplied by the superior, middle, and inferior suprarenal arteries which are branches of the inferior phrenic artery, the abdominal aorta, and the renal artery respectively. Variant suprarenal arteries can be branches of either the abdominal aorta directly or from one of the major branches of the aorta.

Similar to other visceral branches of the abdominal aorta, gonadal arteries and, in particular, the testicular arteries also exhibit anatomical variation. Arched testicular artery is a relatively rare variant and is classified as a type III variety in the Notkovich gonadal arteries classification.^7^ An arched testicular artery ascends after its origin, arches over the renal vein from dorsal to ventral aspect, and then descends further till it reaches the deep inguinal ring. We present herein a case of accessory right renal artery with other vascular variations coexisting in an elderly male cadaver.

## CASE REPORT

**Figure 1:**
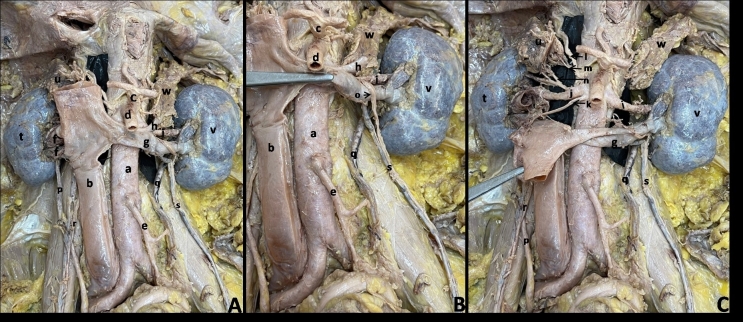
Dissected posterior abdominal wall showing the kidneys and other retroperitoneal structures. **A)** kidneys and suprarenal glands of both sides, **B)** kidney and suprarenal gland of right side, **C)** kidney and suprarenal gland of left side; a- abdominal aorta, b- inferior vena cava, c- celiac trunk, d- superior mesenteric artery, e- inferior mesenteric artery, f- right renal veins, g- left renal vein, h- left suprarenal vein, i- left renal artery, j- right renal artery, k- accessory right renal artery, l- right inferior phrenic artery, m- superior suprarenal artery, n- inferior suprarenal arteries, o- arching left testicular artery, p- right ureter, q- left ureter, r- right testicular vessels, s- left testicular vessels, t-right kidney, u- right suprarenal gland, v- left kidney, w- left suprarenal gland.

Using standard dissection methods, the posterior abdominal wall of a 63-year-old embalmed male cadaver was dissected. The available medical records of the individual were unremarkable. An ARA was observed on the right side originating just distal to the main right renal artery at the level of the upper border of the L2 vertebral body. The ARA ran downward towards the inferior pole of the right kidney, passing anterior to the right renal pelvis. The diameters of the main renal artery and the ARA at origin on the right side were 0.84 cm and 0.26 cm respectively. Two renal veins were observed emerging from the right renal hilum which ran for a very short distance of 1.12 cm before draining into the inferior vena cava almost at right angles (Figure 1A). The middle suprarenal artery was absent on the right side and there were two inferior suprarenal arteries arising from a branch of the main right renal artery (Figure 1B). We also observed an arched testicular artery on the left side which originated from the anterolateral aspect of the abdominal aorta just caudal to the origin of the superior mesenteric artery in a plane posterior to the left renal vein (LRV) (Figure 1C). It ascended up between the LRV and the aorta and appeared lateral to the left suprarenal vein. At the upper margin of the LRV it arched down and ran forward anterior to the LRV until it reached the deep inguinal ring. The right testicular artery had a normal course.

## DISCUSSION

The vascular supply of the retroperitoneal viscera and gonads are derived from the lateral splanchnic branches of the dorsal aorta. According to the ‘Ladder theory’ propounded by Felix, the initial 5^th^ – 9^th^ paired splanchnic branches form a network called the ‘*rete arteriosum urogenitale’*, giving rise to the arteries that supply the retroperitoneal viscera. Any deviation from the normal pattern in this network results in anomalous arteries. During the prenatal period, as the kidney ascends from its initial pelvic position to the postnatal lumbar position, it is successively supplied by the branches arising from regional arteries, most of which degenerate, and only the branch destined to form the future definitive renal artery at the L1-L2 level persists. On the other hand, the gonadal arteries are derived from the persistent branches of the mesonephric arteries around the developing renal pedicle.^1^

On the right side, we observed an accessory renal artery along with absence of the middle suprarenal artery. Accessory renal arteries with increased incidence on the right side are known.^2,3^ In up to 5-20% of cases there are accessory superior and inferior polar arteries^4,5^ and in up to 10% of cases an ARA is described originating directly from the abdominal aorta, just like in this case.^6^ Aberrant polar renal arteries (APRA) constitute an important variety of aberrant renal arteries or ARA and are consistently observed in studies. APRAs are arteries supplying either the superior or inferior poles of the kidneys without passing through the renal hilum. An APRA may be a direct branch of the aorta or it may arise from any of the visceral branches of the aorta located in the vicinity. In 2023, Kumar et al.^8^ described a unique case wherein three arteries were observed supplying the left kidney, two of which were superior and inferior polar arteries, arising from the aorta and the inferior mesenteric artery respectively. In addition to these, the superior polar artery further sprouted a left inferior suprarenal artery. In 2020, Cho and Yoon^9^ described a case with bilateral inferior renal polar arteries that were later confirmed to be inferior segmental arteries after intraparenchymal dissection. Several authors have documented APRA accidentally encountered during other abdominal surgeries.^10,11^ It is important to identify and preserve the APRA as it is a functional terminal artery and its damage could lead to ischemic renal damage and subsequent occurrence of renal failure. El-Sherbiny et al.^12^ demonstrated successful anastomosis of the inferior polar artery to the inferior epigastric artery during renal transplant. The present case can be classified as a ‘perforating renal artery’ as per the 2001 Vilhova et al.^13^ classification system. A perforating renal artery is one that arises from the aorta or from one of its branches, but has an extrahilar entrance and a smaller diameter than that of a segmental renal artery. It would be prudent to call it an accessory renal artery when the diameter of the extra artery is similar to that of the segmental renal artery. The inferior polar arteries are clinically more relevant as they can cause ureteric kinking and subsequently lead to obstruction. Extra-parenchymal ARA is an important consideration before various renal surgeries including renal transplantation. In a very recent article, Funes Hernandez et al.^14^ proposed that renin-dependent hypertension could also be due to presence of ARA, which is often overlooked.

Variations of the arterial supply to the suprarenal gland are clinically relevant for abdominal surgeries including the adrenalectomy operation. Anatomical variations in the inferior suprarenal artery are frequently noted. An absent middle suprarenal artery has been reported in up to 50% of cases on the right side and in 33% of cases on the left side in recent studies.^15^ Concomitant presence of an aberrant gonadal artery with variant suprarenal arteries and increased number of inferior suprarenal arteries, like in the present case, has been described in previous reports.^16^

Kayalvizhi et al.^17^ in 2017 reported in their review that the incidence of ‘arched testicular artery’ or the ‘arched gonadal artery of Luschka’ ranges from 1.7% to 20.3%. The arched testicular artery observed here can be classified as type-III variety of the testicular artery, as per the Notkovich classification system. It resembled the description of two such cases by Naito et al.^18^ This aspect of arching of testicular artery over the LRV is particularly important as it can compress the LRV and impede the venous return not only from the left kidney but also from the left testis. Consequently varicocele, orthostatic proteinuria, and hematuria can occur.^7^ Occurrence of the arched testicular artery has been noted on both sides, but with a predilection for the left side. The reason for the left sided preponderance is however not clear. The greater frequency of arched testicular arteries on the left side may also be one of the factors underlying the higher incidence of left-sided varicocele. There is also a possibility of occurrence of a situation similar to the nutcracker phenomenon with subsequent renal venous hypertension.^19^ To date, an arched testicular artery has not been reported as the cause of nutcracker syndrome, but the possibility of such an etiopathology cannot be ruled out, considering the aberrant anatomy. A short review of recent literature describing arched gonadal arteries is presented in Table 1. Clinicians need to check for the presence of such an arched testicular artery when performing renal surgery.^21^

**Table 1:** Observations made by various authors of occurrences of arched gonadal arteries.

**Authors**	**Population and sample size**	**Right side**	**Left side**
Notkovich^7^	Israeli, 50 cases	4%	16% of cases
Pai et al.^20^	Indian, 34 cases	-	1 case
Naito et al.^18^	Japanese, 2 cases	-	2 cases
Lelli et al.^19^	Spanish, single case	-	1 case
Wadhwa and Soni^21^	Indian, 30 cases	-	1 case
Mamatha et al.^22^	Indian, single case	-	1 case
Grine and Kramer^23^	South African, 123 cases	-	7% of cases
Li^24^	Chinese, single case	-	1 case

All the variations observed in this case could be due to aberrations in development of visceral vessels controlled by a common molecular regulator. Most of these variations are non-lethal, as is evident from the age of the cadaver. However, these variations are of tremendous importance, since the abdominal cavity is frequently explored by surgeons and clinicians for operative procedures and other interventions. Our case reiterates the old dictum that if an anomaly is detected in one vessel, there are chances of encountering other related anomalies as well. Knowledge of these variations is imperative for anatomists, radiologists, and surgeons.

## CONCLUSIONS

Coexistence of multiple vascular anomalies around the kidney is a rare situation. Such variants are prone to iatrogenic damage in the absence of prior knowledge of them. An anomalous arched testicular artery can be the cause of unexplained renal venous hypertension and should be suspected. With the advent of newer minimally invasive and robotic surgeries, ignorance of these variations might lead to an unsuccessful and disastrous outcome.
